# Locus Reference Genomic: reference sequences for the reporting of clinically relevant sequence variants

**DOI:** 10.1093/nar/gkt1198

**Published:** 2013-11-26

**Authors:** Jacqueline A. L. MacArthur, Joannella Morales, Ray E. Tully, Alex Astashyn, Laurent Gil, Elspeth A. Bruford, Pontus Larsson, Paul Flicek, Raymond Dalgleish, Donna R. Maglott, Fiona Cunningham

**Affiliations:** ^1^European Molecular Biology Laboratory, European Bioinformatics Institute, Wellcome Trust Genome Campus, Hinxton, Cambridge CB10 1SD, UK, ^2^National Center for Biotechnology Information, Bethesda, MD 20894, USA, and ^3^Department of Genetics, University of Leicester, Leicester LE1 7RH, UK

## Abstract

Locus Reference Genomic (LRG; http://www.lrg-sequence.org/) records contain internationally recognized stable reference sequences designed specifically for reporting clinically relevant sequence variants. Each LRG is contained within a single file consisting of a stable ‘fixed’ section and a regularly updated ‘updatable’ section. The fixed section contains stable genomic DNA sequence for a genomic region, essential transcripts and proteins for variant reporting and an exon numbering system. The updatable section contains mapping information, annotation of all transcripts and overlapping genes in the region and legacy exon and amino acid numbering systems. LRGs provide a stable framework that is vital for reporting variants, according to Human Genome Variation Society (HGVS) conventions, in genomic DNA, transcript or protein coordinates. To enable translation of information between LRG and genomic coordinates, LRGs include mapping to the human genome assembly. LRGs are compiled and maintained by the National Center for Biotechnology Information (NCBI) and European Bioinformatics Institute (EBI). LRG reference sequences are selected in collaboration with the diagnostic and research communities, locus-specific database curators and mutation consortia. Currently >700 LRGs have been created, of which >400 are publicly available. The aim is to create an LRG for every locus with clinical implications.

## INTRODUCTION

Accurate and unambiguous annotation of disease-causing variants is essential. Central to this is the reference DNA sequence with respect to which a variant is reported. Previously, the lack of accepted and stable reference sequences for variant reporting resulted in the use of different sequences and inconsistency of variant reporting over time. The Locus Reference Genomic (LRG) project addresses these problems (1). Specifically designed for the reporting of diagnostically relevant variants, an LRG provides a stable reference sequence record for a particular genomic locus: genomic DNA, transcript and protein sequences are all included in one record, thereby providing a concise ‘one-stop’ record for variant reporting in all coordinates. The sequences defined by an LRG accession will never change. Mapping of the LRG to current and previous genome assemblies is included in the LRG record, thus overcoming difficulties for the user associated with updates to the genome assembly.

**Figure 1.**
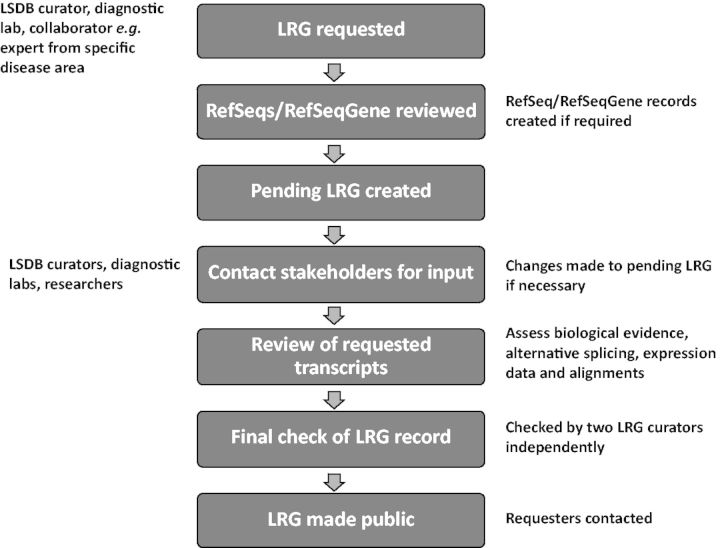
LRG creation process. Steps involved in the creation of each LRG, from initial request to the LRG being made public. All steps are carried out jointly by EBI and NCBI in close collaboration with requesters and collaborators.

The LRG project is a joint National Center for Biotechnology Information (NCBI)/European Bioinformatics Institute (EBI) project, and responsibility for the creation, publication and maintenance of each LRG is shared (Figure 1). This enables integration of data from both NCBI and EBI within the LRG record. All LRG records are available from the LRG website (http://www.lrg-sequence.org/) and are viewable from multiple sites at the NCBI (http://www.ncbi.nlm.nih.gov/refseq/rsg/lrg/) and Ensembl (http://www.ensembl.org/) genome browsers (2).

Adoption of LRGs as the universal reference sequences for variant reporting will facilitate data exchange, ease of comparability and eliminate errors previously caused by imprecise use of reference sequences. This will enable increased submission of disease-causing variants to public databases, improving the accuracy of information available for use in clinical diagnosis, treatment decisions and research.

## CREATION OF LRGS

### Community collaboration

Each LRG is created in collaboration with the community, defined as the research and diagnostic laboratories, locus specific database (LSDB) curators and mutation consortia with expertise in that locus. Working with the community ensures that each LRG record is ideally suited to the demands of variant reporting at a specific locus.

LRG records are created in response to requests from members of the community. Guidelines for making requests are available at http://www.lrg-sequence.org/lrg-request. Once a request is received, a pending LRG is created using the reference sequences specified by the original requester (Figure 1). Each pending LRG record is then reviewed by members of the community for that specific locus. Requesters are asked to provide information on any other members of the community who should be contacted, while additional collaborators are identified from GeneTests (http://genetests.org/), NIH’s Genetic Testing Registry (GTR) (http://www.ncbi.nlm.nih.gov/gtr) and the Leiden University Medical Center LSDB list (http://www.lovd.nl/LSDBs), where available. Additional information on the identification and selection of collaborators is provided on the LRG website (http://www.lrg-sequence.org/lrg-collaborators). Collaborators are asked to review the pending LRG record, provide information on any alternative reference sequences they currently use and are advised on how to switch to reporting variants using LRGs. During the creation process, additional transcripts can be added to the LRG record at the recommendation of a member of the community. Any collaborators with significant involvement in the selection of reference sequences for inclusion in the LRG are asked if they would like to be added as an additional requester. Ultimately, LRGs are created to meet the needs of the community, with final advice and authority resting with them. LRGs can be requested by e-mailing request@lrg-sequence.org.

### LRG fixed section

**Figure 2.**
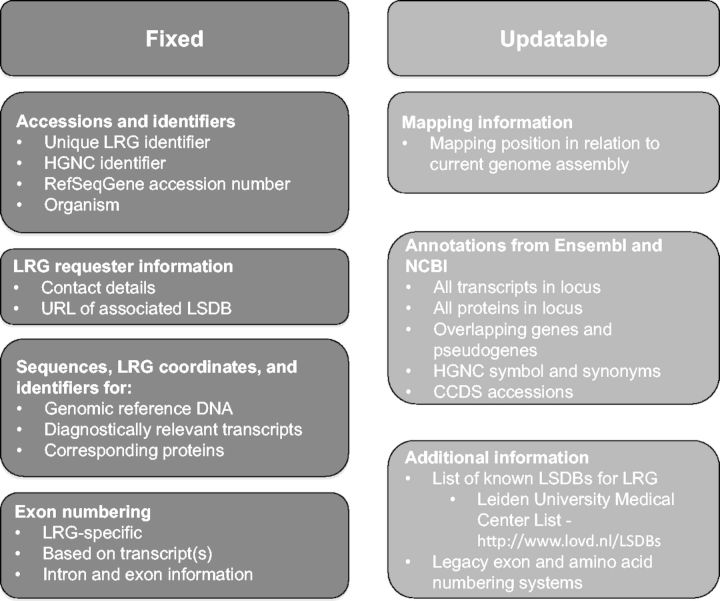
Content and structure of LRG file. The information in each LRG is divided into two sections, fixed and updatable, each with specific content.

LRGs are divided into two main sections: ‘fixed’ and ‘updatable’ (Figure 2). The fixed section contains the core information for the LRG (Figure 2), including requester information, the LRG reference sequences, a unique identifier in the format ‘LRG_[number]’, the HUGO Gene Nomenclature Committee (HGNC) (3) gene identifier and LRG-specific exon numbering.

The LRG reference sequences are composed of a genomic sequence, transcripts required for variant reporting and any corresponding proteins. Sequences submitted for inclusion in the LRG record may be based on RefSeqGene or RefSeq records, or alternative nucleotide sequences in FASTA, GenBank or European Nucleotide Archive format. Most LRG genomic sequences extend 5 kb upstream of the first exon and 2 kb downstream of the last exon, or to the extent necessary to cover all relevant components (i.e. promoters or other regulatory elements). The genomic, transcript and protein sequences of the LRG exactly match those of the corresponding versions of RefSeqGene and RefSeq (4) records (excluding the poly-A tail, which is removed from all LRG transcripts). If RefSeqGene or RefSeq (4) records do not exist to match the sequences requested, the RefSeq curators create such records. Therefore, variant coordinates can easily be translated between these and LRGs. Currently, all LRGs created have been for genic loci; however, they can be created for any locus with clinical implications.

Transcripts for inclusion in the fixed section are initially suggested by the original requester or collaborators. The LRG curators then check NCBI and Ensembl for the most up-to-date biological information data for this locus. For example, they carry out alignments to ensure that all coding sequence with evidence of expression is represented in the transcripts proposed. If there are transcripts with additional sequence and evidence of expression, the curators discuss with the requesters whether these should also be included. As the project aims to reduce ambiguity, only transcripts that are well characterized and are deemed by the community to be essential for variant reporting are included. In practice this means that the majority of LRGs only have one transcript. Limiting the transcripts to those deemed essential by community experts provides guidance to other users with regard to the transcripts that should be used for variant reporting. In rare cases, the collaborators have requested to use an idealized transcript, for example, containing all exons of the gene, as the reporting standard, even though the existence of such a transcript is not supported by biological evidence. Examples of such cases are described in the Supplementary Data. In such cases, the proposed sequences are also reviewed by RefSeq curators and a corresponding RefSeq transcript created.

**Figure 3.**
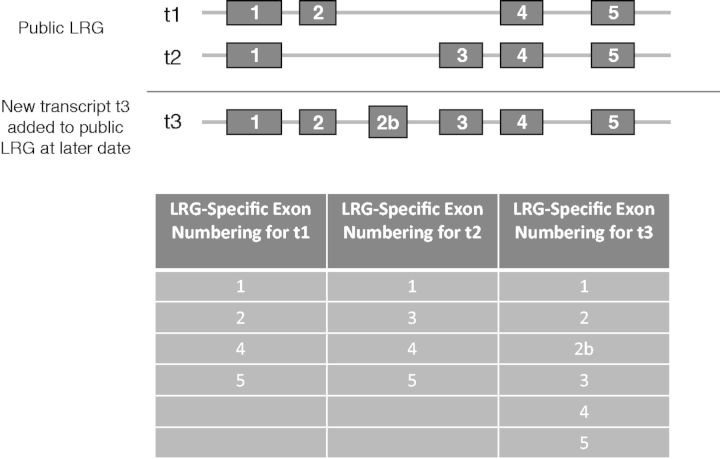
LRG-specific exon numbering scheme. Each LRG contains a fixed LRG-specific exon numbering scheme. Exons of all transcripts included in the fixed section are numbered sequentially 5′–3′ according to their genomic location. This numbering is then applied to individual transcripts. If a transcript is added to the LRG after it has been made publicly available, the existing exon numbering will not change, with any additional exons being assigned numbers in collaboration with the community.

Each LRG has a fixed LRG-specific exon numbering system based on the transcript(s) included in the fixed section. Each distinct exon is numbered consecutively 5′–3′, and then the numbering is applied to individual transcripts (Figure 3). Any transcripts added after the LRG is made public will be assigned exon numbers in collaboration with the community, but will not change the existing LRG-specific exon numbering (Figure 3).

### LRG updatable section

The updatable section contains the mapping of the LRG to the most recent genome build and the most advanced and up-to-date biological knowledge for the LRG region from Ensembl and NCBI. To ensure that each LRG record is kept current, the curators update all existing LRGs twice a year and when a new genome build is released.

The LRG is aligned to the reference genome to determine its mapping coordinates. Each area of contiguous alignment is described separately, along with any differences between the LRG and the reference.

Biological knowledge from NCBI and Ensembl for the LRG region is included in separate sections. Both detail the official names and synonyms of genes, transcripts and proteins contained within the LRG region. Mapping of these sequences in LRG coordinates and any mapping discrepancies are included. Furthermore, there are links from the sequences to the NCBI or Ensembl browsers where additional information, such as expression patterns or biological evidence for each sequence, can be found. The transcript(s) included in the fixed section are also listed in the updatable section, but marked with a note to distinguish them from the other transcripts included in the record.

The final section is for additional data on the LRG locus, namely, a link to the LSDB list (http://www.lovd.nl/LSDBs) for the locus hosted at Leiden University Medical Center. Alternate or legacy numbering systems (exon and amino acid), determined prior to the existence of the LRG and widely used by the community, may also be included in this part of the updatable section to enable easy comparison between different numbering systems.

## NCBI BIOLOGICAL KNOWLEDGE

Annotation provided by NCBI in the updatable section includes RefSeq-related data (accessions, stable sequence identifiers [GIs]), other database identifiers [Consensus CDS identifiers (CCDS) for coding regions (5,6), MIM numbers and GeneIDs] and alternate names and symbols. Any genes partially or completely overlapping the LRG are annotated.

## ENSEMBL BIOLOGICAL KNOWLEDGE

The Ensembl data include protein-coding genes from the Ensembl transcript annotation process (7). All transcripts are based on mRNA and proteins in public scientific databases, and also include the CCDS transcripts. The Ensembl gene set also includes automatically-annotated pseudogenes and non-coding RNAs.

### LRG release

Once the review process is complete, a pending LRG is made public and requesters are notified. From this point, sequences included in the fixed section will not change. Additional transcripts can be added to the fixed section of a public LRG in the future should this be necessary for reporting variants at the locus. If this should happen, the original information in this section, e.g. existing exon numbering, will remain unchanged. LRGs are established to provide a consistent reporting framework. Because of this policy, should an error in an LRG sequence be identified after release, the sequence in the LRG will not be changed. Instead, a second LRG for the locus will be created if the community so requests, with an independent accession number, and the updatable layer of the problematic LRG will be annotated to inform users of the discrepancy.

### Reporting variants on LRGs

All sequences in the LRG fixed section can be used for stable reporting of variants using their stable identifiers. In accordance with HGVS conventions, variant reporting using LRGs as a reference standard is possible in genomic DNA (e.g. LRG_1:g.8463G>C), mRNA (e.g. LRG_1t1:c.572G>C), non-coding RNA (e.g. LRG_163t1:n.5C>T) or protein (e.g. LRG_1p1:p.Gly191Ala) coordinates. Should the need arise in the future to create multiple LRGs for the same locus, these will have different accession numbers, rather than versions of the same accession number. Therefore, this will eliminate the ambiguity caused by versioning in variant reporting. LRG sequences have been endorsed by HGVS (http://www.hgvs.org/mutnomen/) and in the European Molecular Genetics Quality Network best practice guidelines (8). LRGs are also recommended as the reference sequence of choice for LSDBs (9,10). Conversion of existing variant data into LRG coordinates is facilitated using the NCBI Genome Remapping Service (http://www.ncbi.nlm.nih.gov/genome/tools/remap/, Clinical Remap tab). In short, locations or HGVS expressions submitted on a selected genomic assembly or RefSeqGene will be converted to locations on an LRG if one is publicly available for that region.

### Submission of variants

Variants can be submitted to dbSNP (http://www.ncbi.nlm.nih.gov/SNP/) and ClinVar (http://www.ncbi.nlm.nih.gov/clinvar/) via the LRG submission process. This process was ratified at the 2010 Human Variome Project Meeting (11) and is described on the LRG website. A submission template was designed to capture the essential elements to describe genotypic variation and its impact on phenotype and disease. Submissions are loaded to ClinVar. If the variant is not yet represented in dbSNP or dbVar/DGVa (12), then ClinVar reports the variant to the appropriate database at NCBI for assignment of identifiers in those databases. These data are then reported to the submitter and are made publicly available.

### Availability of data

#### LRG website

Each LRG exists as a single file in extensible markup language (XML) format (1). All LRG records (public and pending) are viewable and downloadable on the LRG website (http://www.lrg-sequence.org/), along with all LRG reference sequences in FASTA format and schema documentation (ftp://ftp.ebi.ac.uk/pub/databases/lrgex/docs/LRG_XML_schema_documentation_1_8.pdf). A search box on the LRG home page allows searching by LRG identifier, HGNC symbol, NCBI and Ensembl accession numbers, gene synonym or LRG status. A unique web page for each LRG renders the content of the corresponding XML file into a user-friendly display.

The LRG view on the website includes additional features not available in XML format. For public LRGs, links to Ensembl’s summary LRG page (http://www.ensembl.org/Homo_sapiens/LRG/Summary?lrg=LRG_5) and LRG variation table (http://www.ensembl.org/Homo_sapiens/LRG/Variation_LRG/Table?lrg=LRG_5) are included in the Ensembl annotation section.

#### LRG web services

The LRG web services allow programmatic access to the data for one or more LRGs (see full details on the LRG website: http://www.lrg-sequence.org/web-service). These are based on the web service part of the EB-eye (13) search engine (http://www.ebi.ac.uk/Tools/webservices/services/eb-eye) and use the XML-RPC (http://xmlrpc.scripting.com/default.html) protocol. It is possible, for example, to retrieve the LRG identifiers for a list of HGNC gene symbols, or the genomic sequence for a given LRG. An authentication key is required to access the web service, which can be freely requested from help@lrg-sequence.org.

#### LRG support

LRGs can be viewed in the Ensembl and NCBI genome browsers, usually within 2 months of the LRG being made public (Supplementary Table S1). LRGs are also supported by external software: the Variant Effect Predictor (14) for variation analysis, e.g. from exome sequencing; Mutalyzer (15) for checking sequence variant nomenclature; Alamut (Interactive Biosoftware: http://www.interactive-biosoftware.com/) for variation interpretation; Variobox (16) for annotation, analysis and comparison of human genes; and the the Leiden Open Variation Database (17) DNA variation database system.

### Case studies

Detailed manual curation and extensive collaboration with experts enable the creation of custom-made records that are optimal for reporting variants. Currently >700 records have been created, including some with unique features and for genes with known complexity. Several case studies are described in the Supplementary Data to illustrate how the LRG project has addressed these challenging regions.

### Further information

Communication is an essential part of the LRG project. To facilitate this the LRG website includes a ‘News’ page (http://www.lrg-sequence.org/news) for important developments or schema changes. News items can be received via e-mail by subscribing to the mailing list (e-mail contact@lrg-sequence.org) or via the RSS feed, which publicises changes to the status of LRGs. The LRG website also contains background information on the project and the complete LRG specification, along with instructions on how to request an LRG and submit variants.

### Future developments

The long-term goal of the LRG project is to create an LRG record for every clinically relevant locus. Creation of LRGs is being prioritized according to demand, with LRGs directly requested by the diagnostic community taking precedence. Ongoing collaborations are underway to create LRGs for the genes involved in inherited bleeding and platelet disorders and for the genes included in the United Kingdom National External Quality Assessment Service scheme. LRGs are also being created for genes organized by type of disease as included in commercial diagnostic testing panels, e.g. those available from Cegat (http://www.cegat.de/), GeneDx (http://www.genedx.com/) and the Illumina TruSIGHT panels.

Whenever a new genome assembly is available, and the NCBI and Ensembl databases have been updated, the mapping information and annotation of all LRGs will be updated to the new assembly. Mapping of the LRG genomic sequence to both the current and penultimate assembly will be included. HGVS-compliant variant descriptions based on the LRG will be added to ClinVar to support searching and reporting.

In the future, access to LRG data will also be available through a REST API. Development of this will enable retrieval of LRG data through specific URLs without the need for an authentication key.

## SUPPLEMENTARY DATA

Supplementary Data are available at NAR online, including [18–25].

## FUNDING

The Wellcome Trust [WT095908]; British Heart Foundation [SP/10/10/28431]; European Molecular Biology Laboratory. European Community’s Seventh Framework Programme [FP7/2007-2013] under grant agreement number 200754–the GEN2PHEN project. Work at NCBI is supported by the National Institutes of Health Intramural Research Program and the National Library of Medicine. Funding for open access charge: The Wellcome Trust.

*Conflict of interest statement*. None declared.
